# Pneumatically driven surgical forceps displaying a magnified grasping torque

**DOI:** 10.1002/rcs.2051

**Published:** 2020-01-05

**Authors:** Takuya Iwai, Takahiro Kanno, Tetsuro Miyazaki, Daisuke Haraguchi, Kenji Kawashima

**Affiliations:** ^1^ Department of Biomechanics, Institute of Biomaterials and BioengineeringTokyo Medical and Dental University Tokyo Japan; ^2^ Department of Laboratory for Future Interdisciplinary Research of Science and Technology Institute of Innovative Research, Tokyo Institute of Technology Yokohama Japan

## Abstract

**Background:** Sensing the grasping force and displaying the force for the operator are important for safe operation in robot‐assisted surgery. Although robotic forceps that senses the force by force sensors or driving torque of electric motors is proposed, the force sensors and the motors have some problems such as increase in weight and difficulty of the sterilization.

**Method:** We developed a pneumatically driven robotic forceps that estimates the grasping torque and display the magnified torque for the operator. The robotic forceps has a master device and a slave robot, and they are integrated. In the slave side, the grasping torque is estimated by the pressure change in the pneumatic cylinder. A pneumatic bellows display the torque through a linkage.

**Results:** We confirmed that the slave robot follows the motion of the master, and the grasping torque is estimated in the accuracy of 7 mNm and is magnified and displayed for the operator.

**Conclusions:** The pneumatically driven robotic forceps has the capability in the estimation of the grasping torque and display of the torque. Regarding future work, the usability and fatigues of the surgeons must be evaluated.

## 1 INTRODUCTION

Laparoscopic surgery causes less damage to the patient compared with that caused by open surgery. This surgical method hastens patient recovery and reduces the size of the surgical incision. Although laparoscopic surgery has been adopted for various procedures, it requires increased surgical skill because surgeons have to operate a forceps inserted into a narrow cavity. To clear the space around the organ of interest, a surgeon frequently grasps and pulls up other organs with a grasping forceps. The forceps used in this surgery is long, so insufficient grasping torque is transmitted to the handle and felt by the surgeon.

Control of grasping force has depended on the skill of the surgeon. Surgeons may damage delicate organs such as a liver by excessive force^1^. An effective way to reduce the grasping force is to change the force ratio between the proximal handle and the distal grasper, which is mechanically determined and hard to change conventionally. A bilateral robot forceps enables easy adjustment of the force ratio and may achieve fine control of the grasping force by increasing the force scale in sensitive scenarios.

The design of force‐sensing systems for robotic forceps has been studied by many researchers.^2^, ^3^, ^4^, ^5^, ^6^, ^7^, ^8^, ^9^, ^10^, ^11^, ^12^, ^13^, ^14^, ^15^ Most research uses electromechanical force sensors for these systems, which can be classified by the location of the sensor. One approach is to place a force sensor at the forceps tip, as done by Kuwana et al to directly measure the grasping torque at the tip.^2^ Kim et al measured pulling force and grasping force with three degrees of freedom.^3^ The measurement accuracy of this method is high because the sensor directly measures the force applied to tissues. However, sterilization of sensors is difficult, and the forceps becomes large due to extra wiring.^16^ In other approaches, a force sensor is placed in the forceps shaft or a driving unit. Rosen et al placed a six‐axis force sensor on a laparoscopic tool base.^4^ This approach enables sterilization and downsizing, although the accuracy is lower than that with direct measurement. Regardless of where they are placed, these sensors can increase the cost and size of the device.

Therefore, several methods have been proposed to estimate the grasping force or torque without installing force sensors. Li et al developed a robotic forceps driven by electric motors that estimates the grasping force at the tip by modeling the forceps dynamics.^5^ Tsukamoto et al estimated the grasping torque from the torque of the electric motor driving the gripper.^6^ Frictions and backlashes hide small changes of the external force and highly affect the performance of force estimation. It is necessary to reduce the loss between the actuator and the end‐effector. Electric motor and gears driving a forceps manipulator are usually heavy and result in a large base arm to support the weight. In addition, the reduction mechanism reduces the force transmitted back to the motor from the forceps tip. Moreover, a lot of safety measure is required for surgical robots to prevent electric leakages,^17^, ^18^ especially for motors with higher voltage and current than sensors.

To avoid the problems of these sensing systems, we used pneumatic cylinders to enable direct drive of the robotic forceps.^19^, ^20^, ^21^ The grasping torque is estimated by modeling and compensating the mechanical impedances without the use of motors or electromechanical force sensors.^8^ However, the estimation system generates the grasping force gently, and the response of the force estimation system is slow. Moreover, in the pneumatically driven system, the driving force of the slave depends on the position deviation between the master and the slave. In the grasping task, however, the deviation cannot be large because the master device for the grasping task has the small range of movement. Therefore, the robotic forceps for the grasping task needs the position control system that can generate the large driving force with the small deviation.

The system also has a function of displaying the estimated force to the operator. Display of the grasping torque to the surgeon is important for patient safety,^22^, ^23^, ^24^, ^25^ so many researchers have proposed haptic display systems for surgical robots.^26^, ^27^, ^28^, ^29^ Hu et al developed a haptic device using electric motors that has the ability to display the grasping and push‐pull force.^28^ The device is compact and lightweight, but its cable‐driven mechanism may cause friction force. Kim et al developed a haptic device to display the grasping torque using an electric motor and demonstrated the grasping torque applied to organs can be reduced compared with that without haptic feedback.^27^ This system mainly targets to telesurgery. In the surgical robot, the surgeon teleoperates the slave robot through the master device. On the other hand, a handheld type surgical robot that is not teleoperated has been developed. In the clinical practice, the handheld type has the advantages such as easy to setup, large workspace, and low costs.^30^ Kymerax (Terumo, Japan) is an electrically driven hand‐held forceps.^31^ FlexDex has a mechanical bending joint, which is intuitively operated by a surgeon.^32^ The grasping motions in the existing hand‐held robots are mechanically transmitted, and the adjustment of motion scaling and the force sensing are impossible.

This paper presents a handheld type robotic forceps that is driven by the pneumatic actuator. The robotic forceps has the master device and the slave robot, and these devices are integrated into a handheld robotic forceps. It is lightweight, compact, and low cost compared with the robot for the telesurgery. This robotic forceps makes the risk of the electric accident smaller than the electrically driven robots. In this paper, we propose the position control system for the grasping task that can generate the large grasping torque with the small deviation between the master and the slave. The control system can also estimate the grasping torque at the tip without the force sensors, and the master device displays the magnified grasping torque for the operator. The range of the magnification ratio is confirmed by a stability analysis of the pneumatic bilateral control system. The proposed control system and the estimation performance were confirmed by several experiments.

This paper is organized as follows: The developed robotic forceps is introduced in Section 2. The master device is described in Section 3. In Section 4, the slave robot is described, and the position control system for the grasping task is proposed. The torque estimation method is discussed in Section 5. The bilateral control system is described in Section 6. In Section 7, some experiments are presented, and the performance of this robotic forceps is evaluated. Finally, the conclusion is given in Section 8.

## 2 MASTER‐SLAVE INTEGRATED ROBOTIC FORCEPS

**Figure 1:**
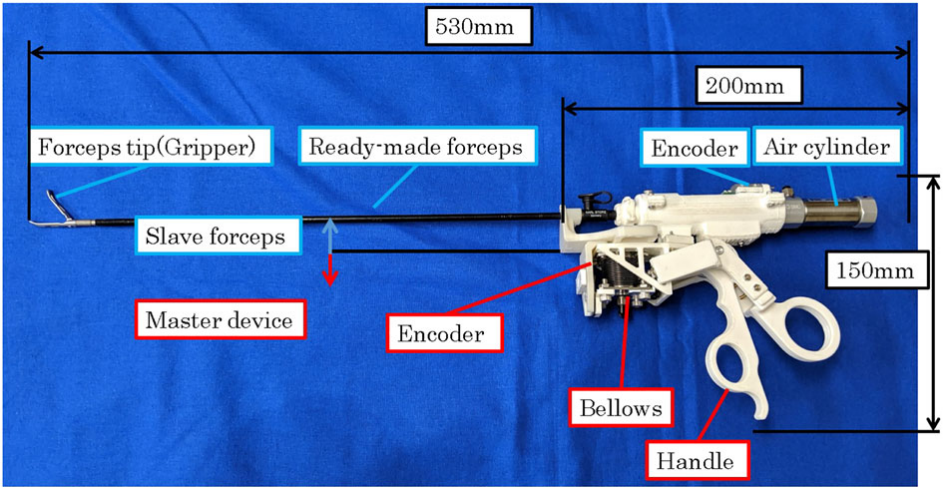
Robotic forceps developed by integrating a slave forceps and master device

**Figure 2:**
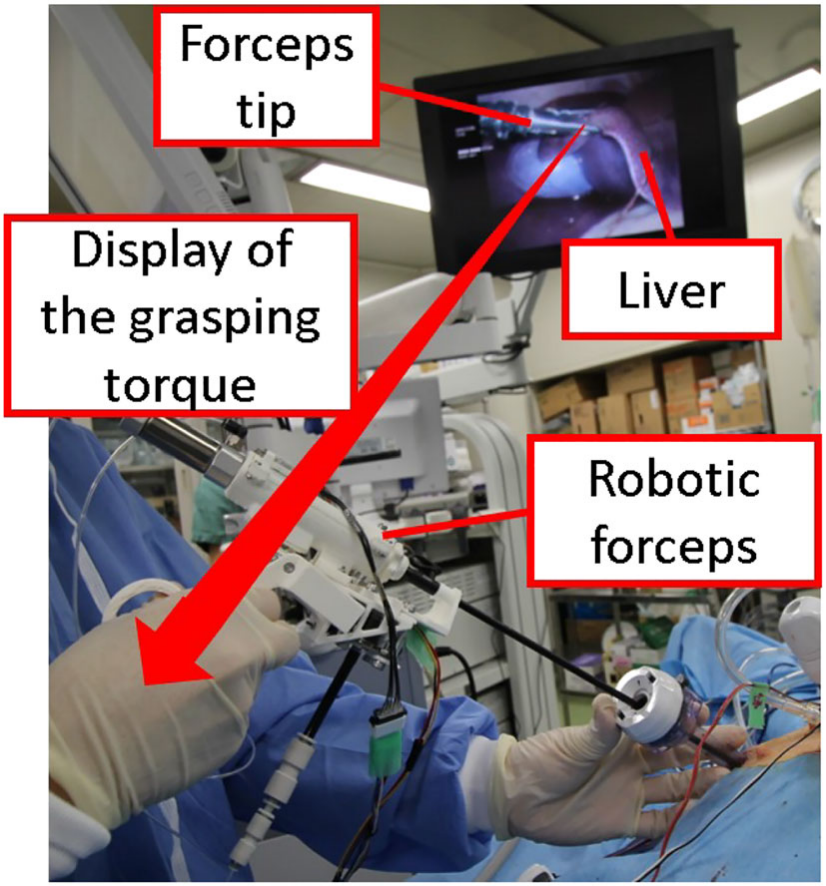
Application of the developed robotic forceps for grasping task

Figure 1 shows the robotic forceps developed by integrating a slave forceps and master device. The robotic forceps can be used to get a large surgical field by grasping the organs as shown in Figure 2. The master device has a handle driven by pneumatic bellows, and the slave forceps is driven by a pneumatic cylinder placed at the posterior end of the forceps shaft. Thought the pneumatic actuators output high power, the risk of the electric leakage is drastically small since the high voltage is not applied to the robot. The operator holds and uses the forceps substantially in the same manner as conventional forceps are used. The pneumatic actuators make the forceps compact and lightweight. The total mass of the developed forceps is 280 g, which is acceptable for long time surgical operation. Even when experts grasped an organ using a robot with force feedback, there was a 0.6 N deviation between the maximum and median grasping force,^25^ which caused no failure of operations.

**Figure 3:**
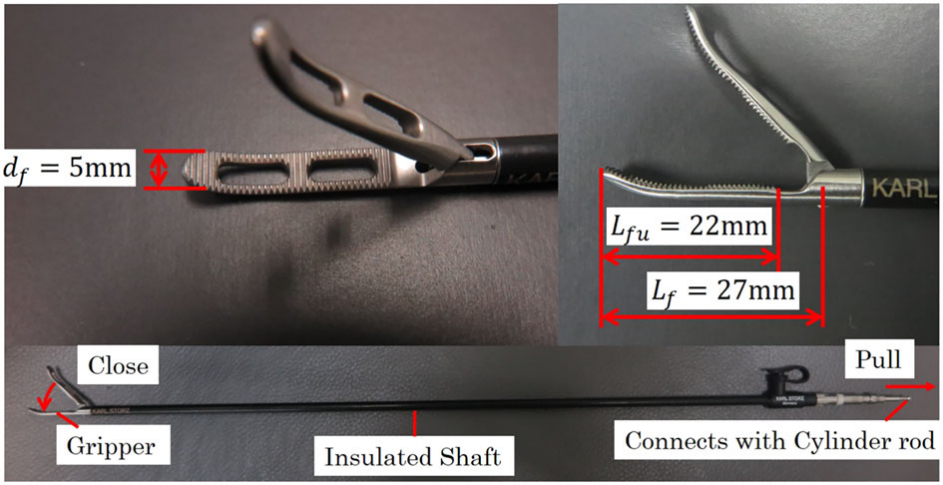
Details of slave grasping forceps

The system is based on the ready‐made laparoscopic grasping forceps tip and shaft shown in Figure 3 (Karl Storz, Croce‐Olmi Grasping Forceps). The forceps tip and pneumatic cylinder can easily be detached for maintenance and sterilization.

## 3 MASTER DEVICE

The master device displays the torque haptically with a pneumatically driven bellows actuator (Irie Koken, material: SUS316L). The bellows used in the device can withstand 0.1 MPa of pressure, and the bellows spring constant is *K*_*b*_=2.5 N/mm. The bellows works with expanding and contracting, and it does not have sliding parts. As the friction in the bellows is negligibly small, the pressure control can realize a precise torque haptic display.

**Figure 4:**
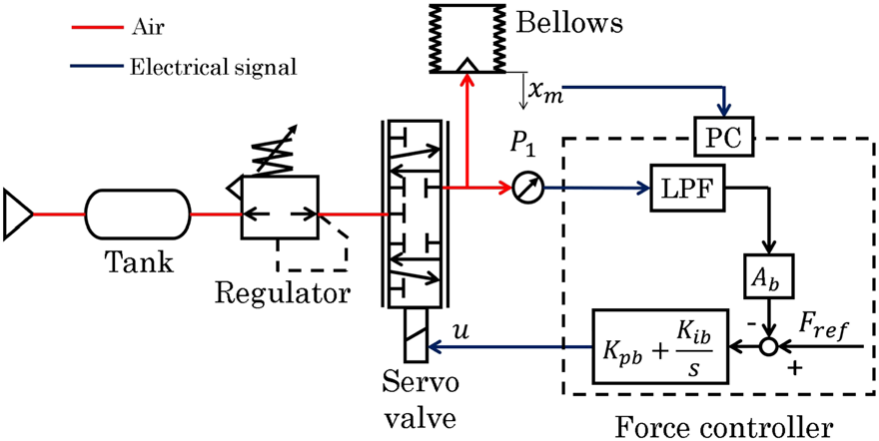
Control system for the master device

Figure 4 shows the pressure control system for the bellows. The bellows expands when it is charged with air pressure, and its position is measured by an encoder (ams, AS5311) installed parallel to the bellows. A proportional‐integral (PI) controller is used to control the bellows pressure.

**Figure 5:**
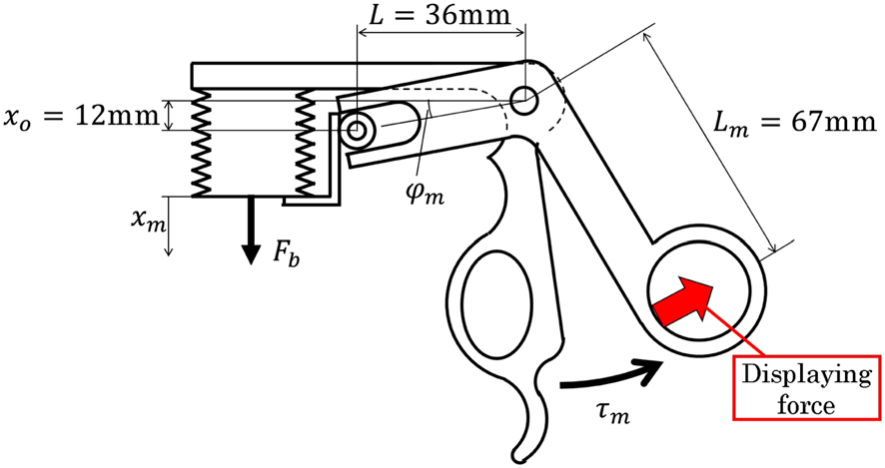
Bellows slider‐lever mechanism in the handle

The PI control gains were determined by trial and error, and the adopted values are shown in Table 1. The pressure is regulated to be 0.1 MPa or less using a servo valve (Festo, MPYE‐5‐M5‐010‐B). The linear motion of the bellows is converted to rotational motion by the slider‐lever mechanism shown in Figure 5. The gripper angle *ϕ*_*m*_ is derived from the bellows displacement *x*_*m*_.

(1) $$\tan\phi_{m}=\frac{x_{o}+x_{m}}{L}.$$

**Table 1:** Proportional‐integral (PI) gain parameters for force control

*K*_*pb*_	0.15 V/N
*K*_*ib*_	0.05 V/N‐s

The relation between the handle torque *τ*_*m*_ and the bellows driving force *F*_*b*_ is calculated using the principle of virtual work.

(2) $$\tau_{m}=\frac{dx_{m}}{d\phi_{m}}F_{b}=\left(1+\frac{{\left(x_{o}+x_{m}\right)}^{2}}{L^{2}}\right)F_{b}L=J_{m}F_{b}.$$

A driving force of 17 N is needed to generate the torque *τ*_*m*_=0.7 Nm at *x*_*m*_=0 mm. The bellows cross‐sectional area *A*_*b*_ is obtained by dividing the driving force by the pressure. Therefore, we selected the initial length and inner diameter of the bellows as 12 and 15 mm, respectively. The handle range of motion was set to 8°, which is similar to that of a conventional forceps. The bellows displacement is 5.2 mm when the handle is fully closed.

## 4 SLAVE FORCEPS

**Figure 6:**
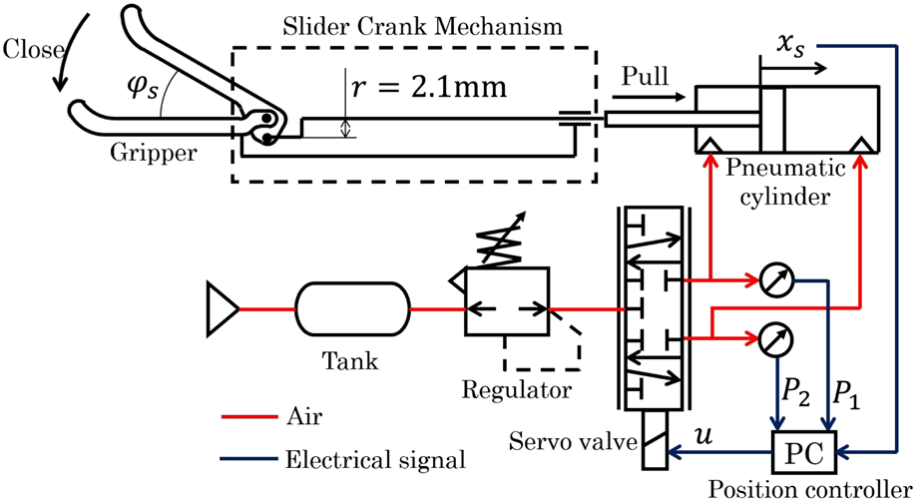
Control system for the slave forceps

Figure 6 shows the pneumatic actuator and control system for the gripper. The ready‐made forceps has a slider‐crank mechanism, and the gripper closes when the slider is pulled. The slider is connected to the cylinder rod and covered by an insulated shaft, which prevents contact between the mechanism and surgical tools, such as a trocar. The maximum opening of the gripper is 50°, which corresponds to a cylinder piston displacement of 1.84 mm.

The gripper opening angle *ϕ*_*s*_ is derived from the cylinder piston displacement *x*_*s*_.

(3) $$x_{s}=r\sin(\phi_{max}-\phi_{s}),$$

where *ϕ*_*max*_ is the maximum opening angle (50°). The gripping torque *τ*_*s*_ and the cylinder driving force *f* are obtained using the principle of virtual work, as follows:

(4) $$\tau_{s}=\sqrt{r^{2}-x_{s}^{2}}f=J_{s}f,$$

where *J*_*s*_ is the Jacobian of the gripping mechanism. According to the other researches, the required force for the grasping organ is about 3 N.^25^ Therefore, we set the maximum torque of the forceps to 80 mNm that is calculated by multiplying 3 N and the gripper length of 27 mm. From (4), *f*=51.9N is needed to generate a gripping torque of 80 mNm with *r*=2.4 mm and *x*_*s*_=1.84 mm. This made it clear that a large force is required to actuate the gripper and that the backdrivable actuator is suitable for estimating the gripping torque. Therefore, we used a low‐friction pneumatic cylinder with an inner diameter of 16 mm, rod diameter of 5 mm, and stroke of 10 mm (SMC, CJ2B16‐10Z). The cylinder can directly generate 100 N of force at a pressure of 0.5 MPa without using a reduction mechanism. The pressure of both chambers in the cylinder, *P*_1_ and *P*_2_, is measured by pressure sensors (SMC, PSE540A‐M5). The cylinder is controlled by a five‐port spool‐type servo valve (Festo, MPYE‐5‐M5‐010‐B). The piston range of travel is 1.84 mm, and its displacement is measured by a magnetic encoder (ams, AS5311; resolution of 2 μm) attached to the cylinder rod. The encoder has minimal noise reduction such as a differential driver.

The pneumatic servo system is nonlinear. However, at the equilibrium point, a linear model is effective for the control design.^19^, ^20^, ^33^ As the travel of the cylinder piston is rather small (1.84 mm), it moves almost at the center of the cylinder. Therefore, we designed the controller with a linear pneumatic servo system model. The equation of motion is given as

(5) $$m\ddot{x_{s}}=A_{1}P_{1}-A_{2}P_{2}-c\dot{x_{s}}.$$

where *m* is the mass of the slave mechanism, *x*_*s*_ is the piston position, *c* is the viscosity coefficient of the slave mechanism, and *A*_1_ and *A*_2_ are the cross‐sectional areas of the cylinder chambers.

The state change of the pressurized chambers is treated as isothermal since the movement is rather slow. Total differentiation of the ideal gas state equation is given as follows:

(6) $$\dot{P_{1}}=\frac{R\theta }{V_{10}}G_{1}-\frac{A_{1}P_{10}}{V_{10}}\dot{x_{s}},$$

(7) $$\dot{P_{2}}=\frac{R\theta }{V_{20}}G_{2}+\frac{A_{2}P_{20}}{V_{20}}\dot{x_{s}},$$

where *R* is the ideal gas constant, *θ* is the temperature of air in the cylinder, *P*_10_ and *P*_20_ are the chamber pressures at equilibrium, *V*_10_ and *V*_20_ are the chamber volumes at equilibrium, and *G*_1_ and *G*_2_ are the mass flow rates for each chamber. The mass flow rate is calculated as follows:

(8) $$G_{1}=\frac{K_{f}}{\sqrt{R\theta }}P_{s}S_{e1},$$

(9) $$G_{2}=\delta \frac{K_{f}}{\sqrt{R\theta }}P_{s}S_{e2},$$

(10) $$\delta =\sqrt{1-{\left(\frac{\frac{P_{a}}{P_{20}}-b}{1-b}\right)}^{2}}\frac{P_{20}}{P_{s}},$$

(11) $$K_{f}=\sqrt{\kappa {\left(\frac{2}{\kappa +1}\right)}^{\frac{\kappa +1}{\kappa -1}}},$$

(12) $$S_{ei}=K_{sv}u,$$

where *P*_*s*_ is the supply pressure, *S*_*e*_ is the effective area of the servo valve, *κ* is the specific heat ratio, b is a ratio between *P*_20_ and *P*_*s*_, *K*_*sv*_ is a proportional constant between the opening of the valve and the input voltage, and *u* is the control signal to the servo valve.

**Figure 7:**
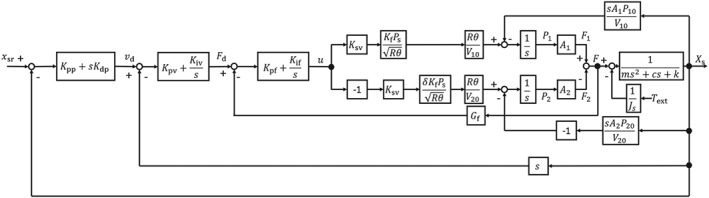
Block diagram of position control system for the slave forceps

Figure 7 is a block diagram of the slave controller design. A cascade approach was adopted for high transparency between the master and the slave and for generation of sufficient gripping torque. The main loop is the position controller, and the minor loops are velocity and force controllers. The reference position is determined to track the master input *x*_*m*_.

(13) $$x_{sr}=\alpha x_{m},$$

where *x*_*sr*_ is the reference of the slave and *α* is a constant value set to $\alpha =\frac{1.84}{5.2}$ so that the gripper and handle both close completely at the same time. The position is controlled by a proportional‐derivative controller, whereas the minor loops use PI control, as shown in Figure 7. The control gains were determined by trial and error, and the adopted values are shown in Table 2.

**Table 2:** Proportional‐integral‐derivative (PID) gain parameters for slave forceps control

*K*_*pp*_	15 1/s
*K*_*dp*_	0.3
*K*_*pv*_	0.1 Ns/mm
*K*_*iv*_	30 N/mm
*K*_*pf*_	1.5 V/N
*K*_*if*_	0.2V/Ns

The operability deteriorates when the position deviation between the master and the slave is large, so we evaluated the influence of the external torque *τ*_*ext*_ applied to the gripper on the position deviation. The position deviation *e*_*p*_=*x*_*sr*_−*x*_*s*_ is calculated as follows for step inputs of *x*_*sr*_ and *τ*_*ext*_.

(14) $$e_{p}=\left(1-\frac{K_{A}G_{F}G_{V}G_{P}}{G_{ch}}\right)\frac{x_{sr}}{s}+\frac{s+K_{A}G_{F}G_{f}}{G_{ch}}\frac{\tau_{ext}}{sJ_{s}},$$

(15) $$G_{ch}=(ms^{2}+cs)(s+K_{A}G_{F}G_{f})+K_{A}G_{F}G_{V}(G_{P}+s)+sK_{B},$$

where *G*_*P*_ is the PD controller, *G*_*V*_ and *G*_*F*_ are the PI controller, and *K*_*A*_ and *K*_*B*_ are given as

(16) $$K_{A}=K_{f}\sqrt{R\theta }P_{s}K_{sv}\left(\frac{A_{1}}{V_{10}}+\delta \frac{A_{2}}{V_{20}}\right)K_{B}=\frac{P_{10}A_{1}^{2}}{V_{10}}+\frac{P_{20}A_{2}^{2}}{V_{20}}.$$

Since the grasping forceps are mainly used to grasp tissue for periods of time, the system can be regarded as a steady state. Therefore, *e*_*p*_ is given from the final‐value theorem.

(17) $$e_{p}{|}_{t\to \infty }=\underset{s\to 0}{\lim}se_{p}=0.$$

It is clear from (17) that the steady‐state error becomes 0 mm. The actual positions of the master and slave coincide with those determined by the cascade controller.

## 5 ESTIMATION OF GRASPING TORQUE

The input force *F* to the pneumatic cylinder is derived from the block diagram in Figure 7 as follows:

(18) $$\begin{array}{ccc} F= & A_{1}P_{1}-A_{2}P_{2} & \\ = & \frac{K_{A}G_{F}G_{V}G_{P}(ms^{2}+cs)}{G_{ch}}x_{sr}+\frac{sK_{B}+K_{A}G_{F}G_{V}(G_{P}+s)}{G_{ch}}\frac{1}{J_{s}}\tau_{ext}. \end{array}$$

The force *F* at a steady state is given as

(19) $$F{|}_{t\to \infty }=\underset{s\to 0}{\lim}sF=\frac{1}{J_{s}}\tau_{ext}=\frac{1}{J_{s}}\tau_{ext},$$

(20) $$\tau_{ext}=J_{s}f.$$

It is clear from (20) that the gripping torque *τ*_*ext*_ can be estimated from the piston position and the generated force of the pneumatic cylinder. The force *f* is obtained from the measured pressures as shown in (18). The position of the piston *x*_*s*_ is measured by the encoder.

The driving force is increased by the viscosity of the slave mechanism during the motion. Therefore, the viscosity is compensated by the following equation.

(21) $$\tau_{ext}=J_{s}\left(f-c\dot{x_{s}}\right).$$

The viscous friction, *c*=8.0 Ns/mm, was determined experimentally. To display the estimated gripping torque to the operator, the target value of handle torque is given by the following equation:

(22) $$\tau_{m}=\beta \tau_{ext},$$

where *β* is a scaling ratio to magnify the haptic display force. The operator can more easily feel a small force when *β* is increased.

The force *F*_*b*_ is calculated from (2) using *τ*_*m*_. This force is given as the reference input to the master device.

## 6 BILATERAL CONTROL SYSTEM

**Figure 8:**
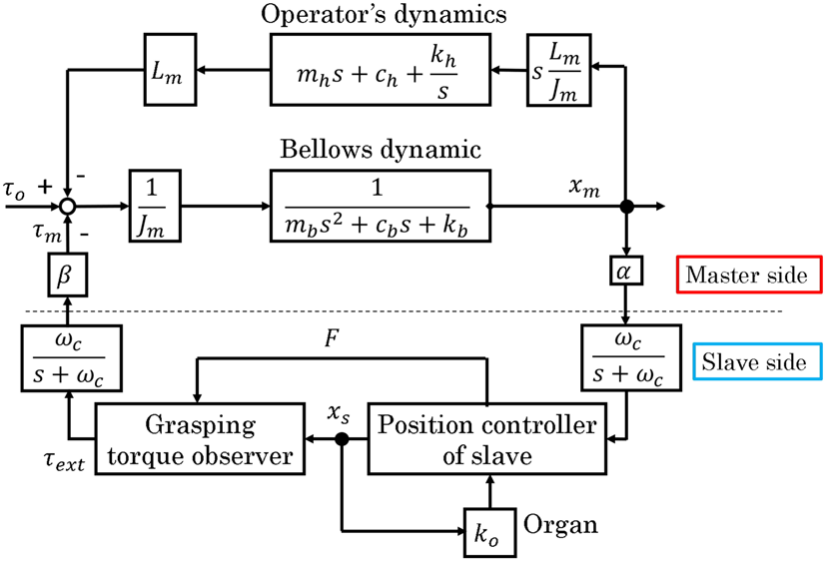
Block diagram of bilateral control system

A force‐reflecting bilateral control was implemented in the developed robotic forceps. Figure 8 shows the block diagram for the system. In the control system, since the communication between the master and slave is executed by memory access, the communication delay can be ignored. The operator grips the handle to generate torque *τ*_*o*_ and the bellows contracts. The bellows deformation *x*_*m*_ is given as a reference input to the slave side of the controller. The *x*_*m*_ is filtered by a low‐pass‐filter to cancel the noise. Here, the scaling factor *α* can be multiplied by *x*_*m*_ to change the scaling ratio. When the forceps grasps tissue, the grasping torque *τ*_*ext*_ is estimated from the driving force of the pneumatic cylinder, *F*. Then, the estimated torque is amplified by *β*, transmitted to the master force controller, and displayed to the operator as torque in the handle, as shown in Figure 8.

**Figure 9:**
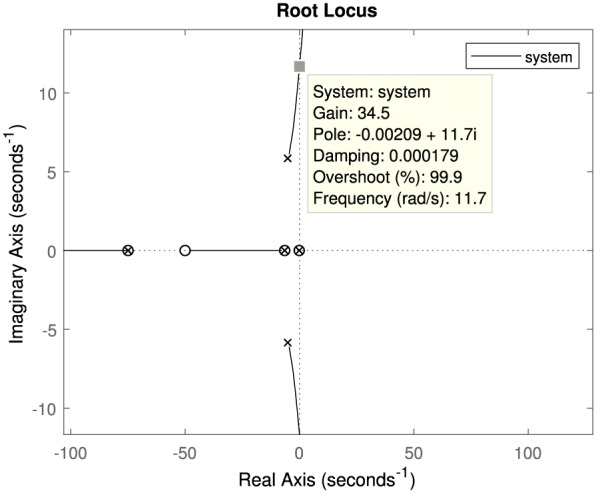
Root Locus of the bilateral control system. In this figure, the gain indicates *β*

The *α* and *β* affect the stability of the bilateral control system. The control system is analyzed by using *MATLAB* to see the effect of the scaling factors. In typical laparoscopic surgery, the motion scaling from the master to the slave is set under 1.0.^34^ The scaling factor *α* is 1.84/5.2 considering the motion range of the master and the slave grippers. Since the motion range of grippers is small compared with translational motions, changing the scaling by software is unpractical, and we fixed the *α* in this analysis. Therefore, we deal with the *β* the feedback gain of the bilateral control system. The bellows is assumed as a mass‐spring‐damper system. The impedance parameters are determined such that the damping ratio is 0.7. The dynamics of the operator is regarded as a mass‐spring‐damper system, and the parameters are set to a literature value.^35^ Table 3 shows the parameters for the stability analysis. The parameters are given by the catalog spec. The organ is assumed to be a spring, and the spring constant is determined by the in vivo experiment. The input is *τ*_*o*_, and the output is *x*_*m*_. Figure 9 shows the root locus. In Figure 9, each lines are the root locus, and all the markers are the roots of this system. It is found from Figure 9 that the system is stable if the *β* is less than 34.5.

**Table 3:** Parameters of the bilateral control system

*m*	0.02 kg
*m*_*b*_	0.05 kg
*c*_*b*_	15.0 Ns/m
*k*_*b*_	2300 N/m
*m*_*h*_	17.5 kg
*c*_*h*_	175 Ns/m
*k*_*h*_	175 N/m
*K*_*o*_	60.2Nm/m
*K*_*f*_	0.121
*K*_*sv*_	6.28×10^−7^ *m* ^*2*^/V
*θ*	287 K
*P*_10_	277 kPa
*P*_20_	250 kPa
*P*_*s*_	500 kPa
*ω*_*c*_	70 Hz

## 7 EXPERIMENTS

The performance of the developed robotic forceps was evaluated experimentally.

First, the performance of the position control system was confirmed. Then, the torque estimation ability of the forceps was confirmed from static and bilateral experiments.

### 7.1 Experiment for the position control system

**Figure 10:**
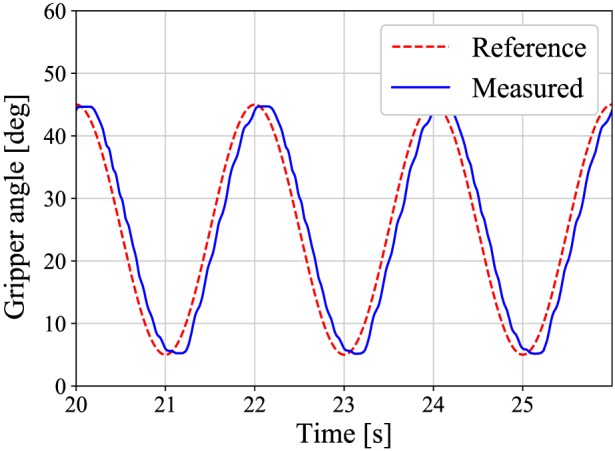
A response of the position control system at frequency of 0.5 Hz

**Figure 11:**
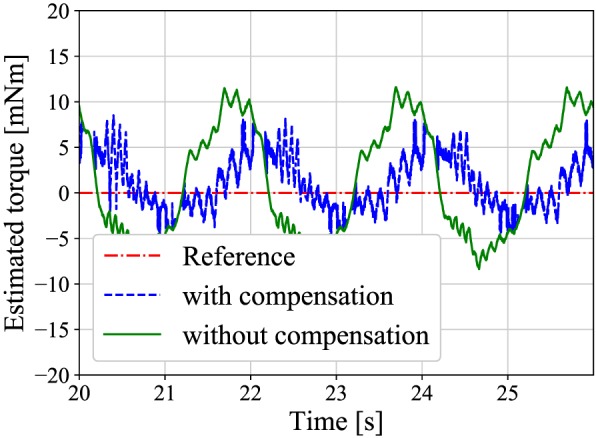
A comparison between the estimated torques with and without the compensation

First, the experiment of the position control was conducted to confirm that the slave follows the reference angle and that the viscosity compensation is effective. A sinusoidal reference position with an amplitude of 20°, an offset of 25°, and a frequency of 0.5 Hz was input into the slave. The gripper grasps no obstacles during the position control. Figure 10 shows the result of the position control, and Figure 11 shows the estimated torques that are compensated by (21) and not compensated. As shown in Figure 10, the slave follows the reference wave. From Figure 11, the amplitude of the compensated torque is smaller than the other. The root‐mean‐square‐error (RMSE) of the compensated and not compensated torques are apploximately 3 and 6 mNm, respectively.

### 7.2 Experiment for static torque estimation

**Figure 12:**
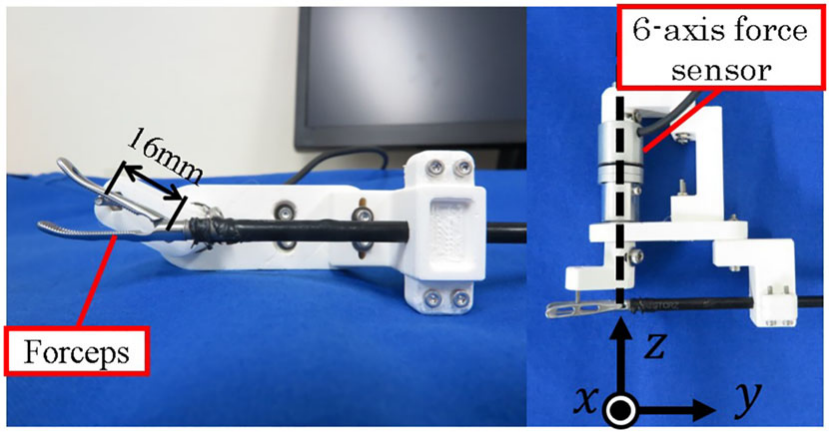
Experimental setup for torque measurement

In the following experiments, a six‐axis force sensor was grasped by the forceps, and the measured and estimated torques were compared. As shown in the right side of Figure 12, the Z‐axis of the six‐axis force sensor and the center of rotation of the gripper were set to be coaxial. The gripper grasped the handle of the sensor 16 mm from the rotation center. The reference of the grasping torque was increased from 10 to 80 mNm. The angle of the gripper was set at approximately 30°. The estimated torques were compared with the gripping torques measured directly by the force sensor.

**Figure 13:**
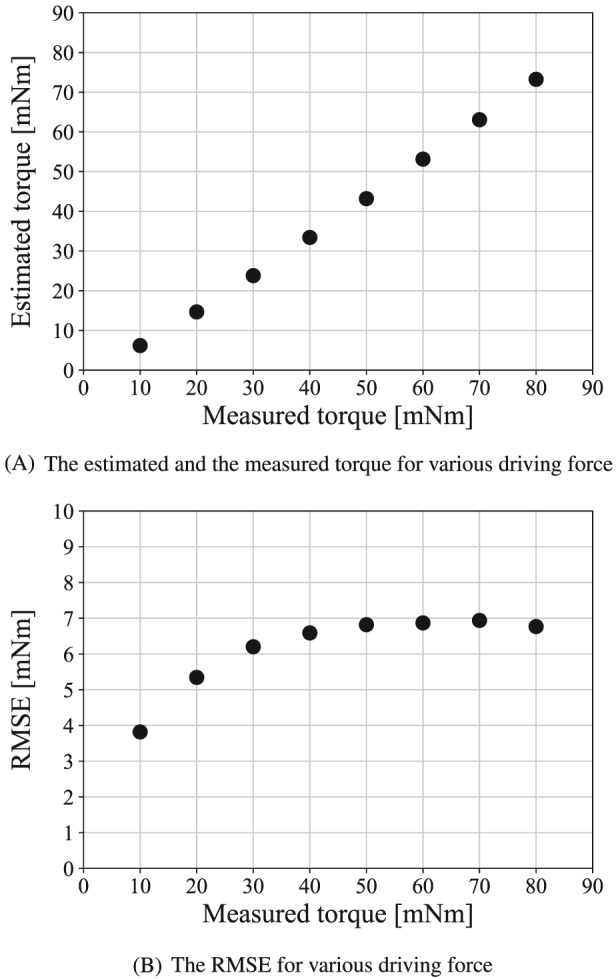
Experimental result for static torque estimation

Figure 13A shows the experimental results. The vertical axis shows the estimated torque, and the horizontal axis shows the measured torque. The RMSE between the measured and estimated torques is shown in Figure 13B.

Figure 13A shows that the measured and estimated torques increase linearly. It is confirmed that the torque estimation can recognize the amplitude of the grasping torque. Figure 13B shows that the larger the driving force is, the larger the estimation error is. The maximum estimation error is 7 mNm. This is due to the static friction force of the slave mechanism. In the practical situation, the deviation of the grasping force is approximately 0.5 N.^25^ The force of 0.5 N at the forceps tip equals to the grasping torque of 14 mNm. Therefore, the error of 7 mNm is smaller than the human ability, and it is acceptable.

**Figure 14:**
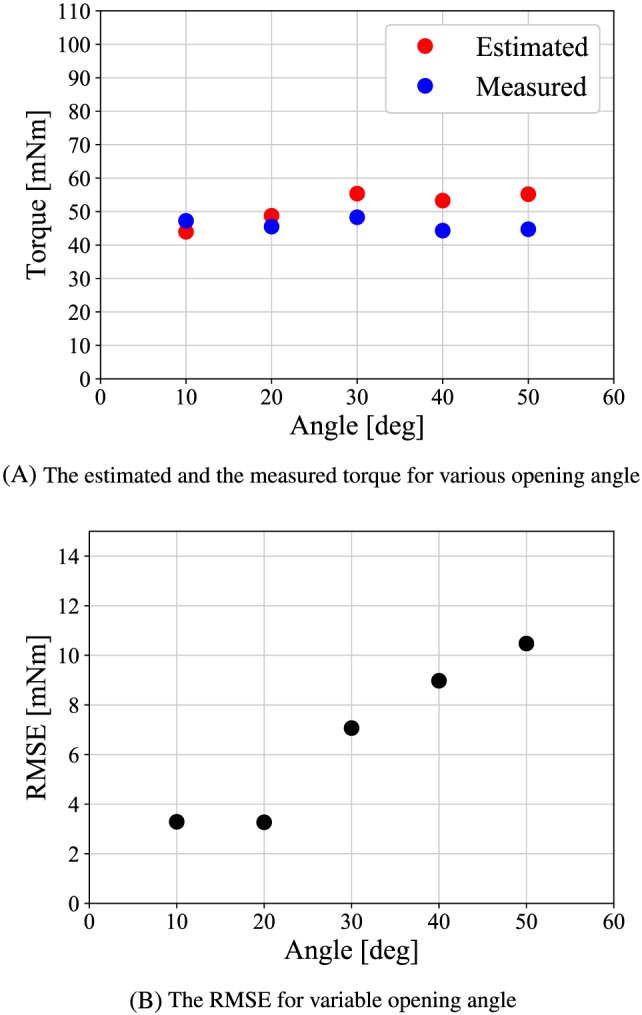
Experimental result for static estimation at various opening angle

Next, the angle of the gripper was varied from 10° to 50° increments to see whether the estimation accuracy depends on the gripper angle. The reference of the grasping torque is set to a constant value of 56 mNm. Figure 14A shows the experimental results. The vertical axis shows the torque, and the horizontal axis shows the gripper opening angle. The RMSE between the actual and estimated torques is shown in Figure 14B.

Figure 14A,B shows that the RMSE becomes smaller at small angle. It is occured because the jacobian of the forceps at the small angle increases the backdrivability of the slave mechanism.

### 7.3 Experiment for bilateral control

**Figure 15:**
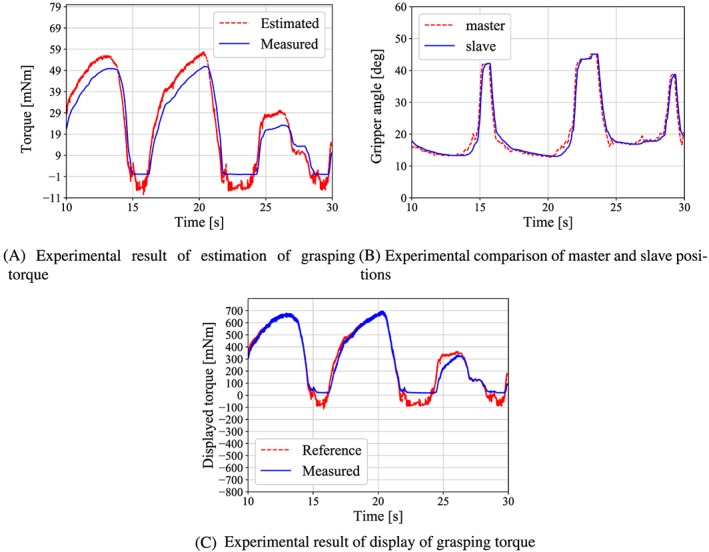
Results of the bilateral control experiment

Bilateral control experiments were conducted to confirm the effectiveness of the developed forceps. A reference position was input by the operator's gripping motion. The gripper grasps the force sensor at the angle of 25°. The resulting torque was measured, and then the positions of the master handle and slave gripper were compared. The *β* is set to 12 that is determined by surgeon's opinions. Figure 15A shows the experimental results for the estimated and measured grasping torques, and Figure 15B shows the positions of the master and slave. The comparison results of the estimated grasping torque and the calculated handle torque are shown in Figure 15C. The handle torque was calculated by applying (2) to the measured values of *x*_*m*_ and *F*_*b*_.

Figure 15A indicates that the torque is estimated without delay. The RMSE between the estimated and the measured torque is approximately 6 mNm. Figure 15B shows that the position of the slave coincides with that of the master as given by (17), even when the grasping torque was concentrated at the forceps tip. It is clear from Figure 15C that the operator feels the grasping torque because the trends of both torques are in good agreement overall and also the displayed torque is 12 times larger than the estimated torque.

## 8 CONCLUSION

In this paper, we described the development of a master‐slave robotic forceps with pneumatic actuators. The developed robotic forceps can prevent the surgeons from grasping the organ with large grasping torque. The weight of the proposed forceps is heavy compared with the conventional grasping forceps. However, the weight of 280 g is similar to commercial needle driver; thus, the weight is acceptable in practice. Also, in the pneumatically driven system, the risk of the electric accident is greatly smaller than the electrically driven forceps. The pneumatic actuator provides an estimation of the grasping torque. The robotic forceps estimates the grasping torque and displays it in the handle at any magnification ratio selected by the operator. The magnification ratio can be set up to 34.5, and the grasping task was carried out at the ratio of 12. Experimental results indicate that the robotic forceps can estimate the grasping torque with an error of 7 mNm and haptically display a magnified torque precisely to the operator.

Regarding future work, evaluating in detail the effect of the displaying the magnified grasping torque, the usability, fatigues of the surgeons must be conducted.
